# Tunable antireflection from conformal Al-doped ZnO films on nanofaceted Si templates

**DOI:** 10.1186/1556-276X-9-192

**Published:** 2014-04-26

**Authors:** Tanmoy Basu, Mohit Kumar, Pratap Kumar Sahoo, Aloke Kanjilal, Tapobrata Som

**Affiliations:** 1 Institute of Physics, Sachivalaya Marg, Bhubaneswar 751005, India; 2 National Institute of Science Education and Research, Sachivalaya Marg, Bhubaneswar 751005, India; 3 Department of Physics, School of Natural Sciences, Shiv Nadar University, Gautam Budh, Nagar, Uttar Pradesh 203207, India

**Keywords:** Ion beam-induced nanopatterning, Silicon, Aluminum-doped zinc oxide, Sputter deposition, Antireflection property

## Abstract

Photon harvesting by reducing reflection loss is the basis of photovoltaic devices. Here, we show the efficacy of Al-doped ZnO (AZO) overlayer on ion beam-synthesized nanofaceted silicon for suppressing reflection loss. In particular, we demonstrate thickness-dependent tunable antireflection (AR) from conformally grown AZO layer, showing a systematic shift in the reflection minima from ultraviolet to visible to near-infrared ranges with increasing thickness. Tunable AR property is understood in light of depth-dependent refractive index of nanofaceted silicon and AZO overlayer. This improved AR property significantly increases the fill factor of such textured heterostructures, which reaches its maximum for 60-nm AZO compared to the ones based on planar silicon. This thickness matches with the one that shows the maximum reduction in surface reflectance.

**PACS:**

81.07.-b; 42.79.Wc; 81.16.Rf; 81.15.Cd

## Background

Aluminum-doped ZnO, a transparent conducting oxide (TCO), is becoming increasingly popular as window layer and top electrode for next-generation highly efficient silicon-based heterojunction solar cells [1-4]. An essential criterion to enhance the efficiency of silicon-based solar cells is to reduce the front surface reflection. However, commercial silicon wafers show surface reflection of more than 30% [5]. Such a high level of reflection can be minimized by growing a suitable antireflection (AR) coating, preferably in the form of a TCO. On the basis of thin film interference property, these dielectric coatings reduce the intensity of the reflected wave. However, this approach needs a large number of layers to achieve well-defined AR properties. In addition, coating materials with good AR properties and low absorption in the ultraviolet (UV) range are rare in the literature. An alternative to the lone usage of dielectric coating is therefore required which can overcome some of these difficulties.

An optimal antireflective surface should contain subwavelength features where the index matching at the substrate interface leads to improved AR performance. For instance, by using a surface texture on TCO (e.g., AZO) [6] and/or Si substrate [7], one can govern the light propagation and in turn the AR property due to the formation of graded refractive index [8,9]. In particular, for solar cell applications, a patterned AZO film on a flat silicon substrate shows a significant decrease in average reflectance up to 5% [10], whereas a thick AZO layer on silicon nanopillars is found to give an overall reflectance of approximately 10% [7]. In the latter case, a higher photocurrent density was achieved (5.5 mA cm^-2^) as compared to AZO deposited on planar silicon (1.1 mA cm^-2^). It is, therefore, exigent to have more control on pattern formation and optimization of AZO thickness to achieve improved AR performance.

Majority of the patterning processes are based on conventional lithographic techniques [11]. As a result, these are time-consuming and involve multiple processing steps. On the other hand, low-energy ion beam sputtering has shown its potential as a single-step and fast processing route to produce large-area (size tunable), self-organized nanoscale patterned surfaces [12] compatible to the present semiconductor industry, and thus may be considered to be challenging to develop AR surfaces for photovoltaics.

In this letter, we show the efficacy of one-step ion beam-fabricated nanofaceted silicon templates [13] for growth of conformal AZO overlayer and correlate its thickness-dependent (in the range of 30 to 90 nm) AR property. We show that growth of an optimum AZO overlayer thickness can help to achieve maximum reduction in surface reflectance. As a possible application of such heterostructures in photovoltaics, photoresponsivity of AZO deposited on pristine and faceted Si has also been investigated. The results show that by using nanofaceted silicon templates, it is possible to enhance the fill factor (FF) of the device by a factor of 2.5.

## Methods

**Figure 1:**
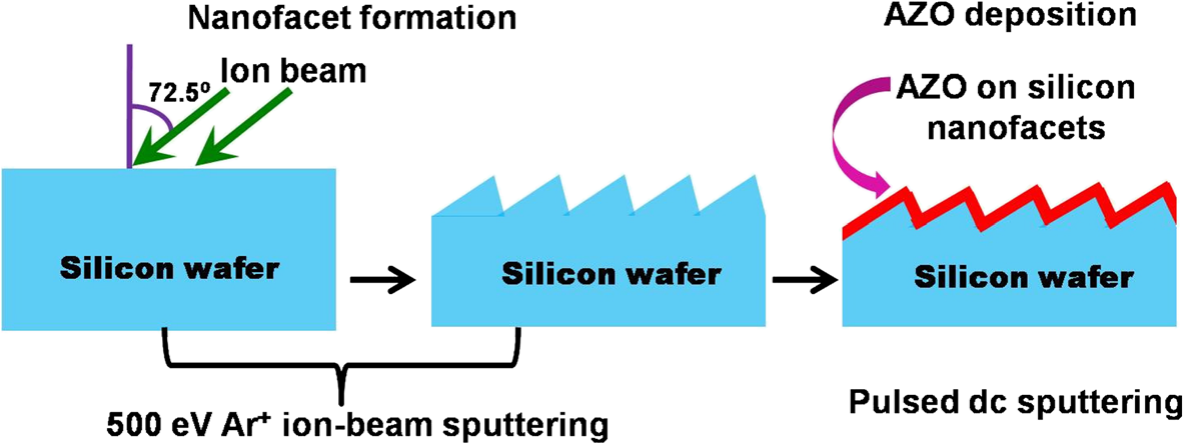
Flow chart for ionbeam fabrication of nanofaceted Si followed by conformal growth of AZO films.

The substrates used in the experiments were cut into small pieces (area 1 × 1 cm^2^) from a *p*-Si(100) wafer. An ultrahigh vacuum (UHV)-compatible experimental chamber (Prevac, Rogów, Poland) was used which is equipped with a five-axes sample manipulator and an electron cyclotron resonance (ECR)-based broad beam, filamentless ion source (GEN-II, Tectra GmbH, Frankfurt, Germany). Silicon pieces were fixed on a sample holder where a sacrificial silicon wafer ensured a low-impurity environment. The beam diameter and the fixed ionflux were measured to be 3 cm and 1.3 × 10^14^ ions cm^-2^ s^-1^, respectively. Corresponding to this flux of 500-eV Ar^+^ ions, the rise in sample temperature is expected to be nominal from room temperature (RT). Experiments were carried out at an ion incidence angle of 72.5° (with respect to the surface normal) and for an optimized fluence of 3 × 10^18^ ions cm^-2^ to fabricate nanofaceted silicon templates. The substrates were immediately transferred to the sputtering chamber (base pressure 3 × 10^-7^ mbar) for growth of AZO overlayers. A commercial (purity 99.99%) target (Testbourne, Basingstoke, UK) composed of ZnO/Al_2_O_3_ (2 wt.%) was used for deposition of AZO films at RT and at an optimized angle of 50°. During film growth, the argon gas flow rate was maintained at 30 sccm, resulting in the working pressure of 5 × 10^-3^ mbar. The distance from the sample to the target was 10 cm, and the pulsed dc power was maintained at 100 W. Figure 1 shows a schematic representation of the process flow towards the synthesis of nanofaceted silicon, and the growth of AZO overlayer on the same thicknesses (in the range of 30 to 90 nm) was measured by using a surface profilometer (XP-200, Ambios Technology, Santa Cruz, CA, USA). Field emission scanning electron microscopy (SEM) (CarlZeiss, Oberkochen, Germany) was employed to study the sample microstructures and to ensure the uniformity of the structures. Sample morphologies were studied by using an atomic force microscope (AFM) (MFP3D, Asylum Research, Santa Barbara, CA, USA) in the tapping mode. AFM images were analyzed by using WSxM and Gwyddion softwares [14,15]. Crystallinity and phase identification of the films were investigated by X-ray diffraction (XRD) (D8-Discover, Bruker, Karlsruhe, Germany), whereas the optical reflectance measurements were carried out by using a UV-Vis-NIR spectrophotometer (3101PC, Shimadzu, Kyoto, Japan) in the wavelength range of 300 to 800 nm with unpolarized light. A specular geometry was used for these measurements where the incident light fell on the target at an angle of 45° with respect to the surface normal. Photoresponsivity studies were performed using a spectral response system (Sciencetech, Ontario, Canada) under air mass 0 and 1 sun illumination conditions in the spectral range of 300 to 800 nm. The incident light power was measured with a calibrated silicon photodiode at wavelengths below 1,100 nm, and the spectra were normalized to the power.

## Results and discussion

**Figure 2:**
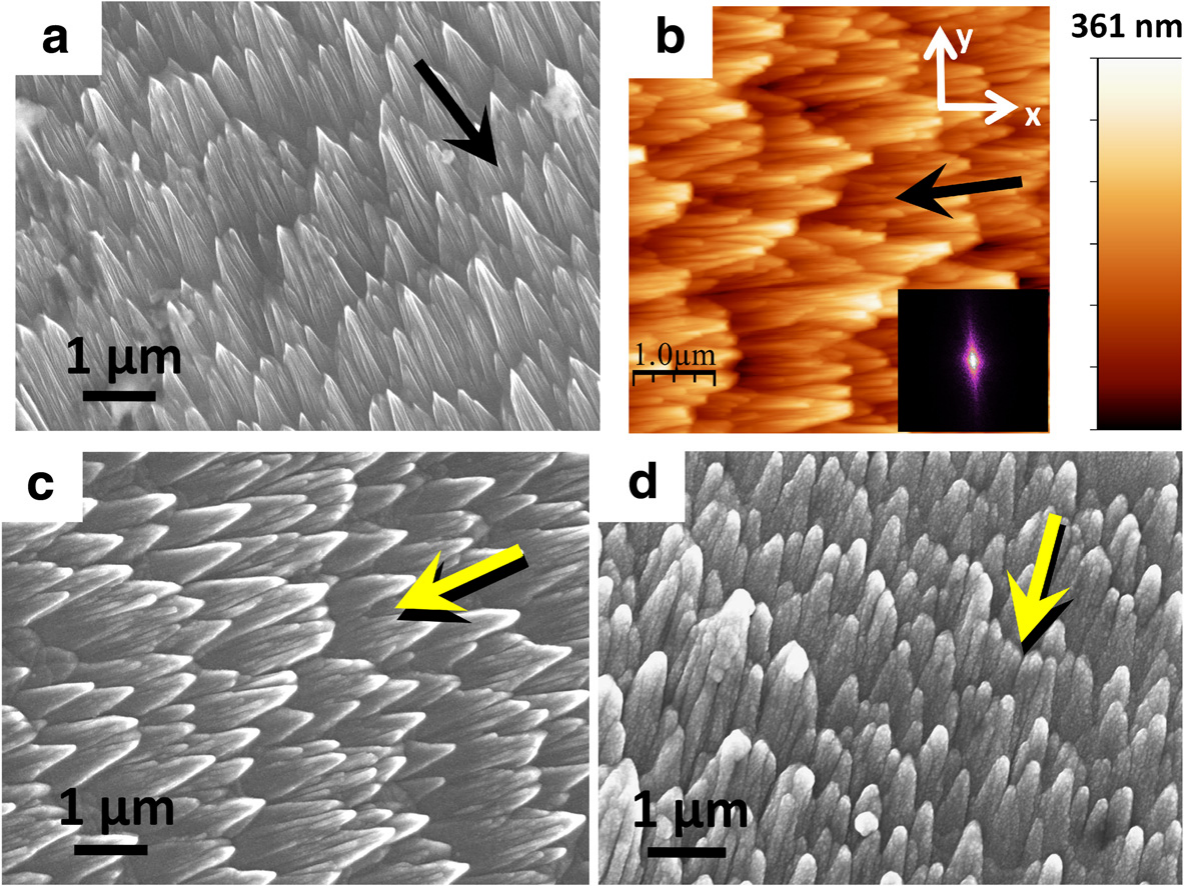
**Plan-view SEM images. (a)** Faceted Si nanostructures. **(b)** AFM topographic image where inset shows the 2D FFT. **(c, d)** After growing AZO films on nanofaceted Si having thicknesses of 30 and 75 nm, respectively. The black arrows indicate the direction of ionbeam bombardment, whereas the yellow arrows represent the direction of AZO flux during sputter deposition.

Figure 2a shows the SEM image of a typical ion beam-fabricated silicon template under consideration, manifesting distinct faceted morphology with striations on its walls. Corresponding AFM image, shown in Figure 2b, indicates that the Si facets are oriented in the direction of incident ion beam. Analysis of this image provides rms roughness value of 52.5 nm, whereas the average silicon facet height turns out to be approximately 180 nm [14]. Two-dimensional (2D) fast Fourier transform (FFT) image, obtained by using Gwyddion software, is depicted in the inset of Figure 2b where a clear anisotropy in the surface morphology is visible along the direction perpendicular to the ion beam projection onto the surface [15]. One-dimensional (1D) power spectral density as well as autocorrelation function (not shown here), along both *x* and *y* directions, does not reveal any periodicity in the case of Si nanofacets. This corroborates well with the absence of any distinct spots symmetrically spaced about the central spot seen in the FFT image. Figure 2c,d depicts the morphologies of nanofaceted Si templates after deposition of AZO overlayers having nominal thicknesses of 30 and 75 nm, respectively. Both these images clearly manifest the conformal growth of AZO on Si facets, albeit with increasing AZO thickness, sharpness of the facets reduces and they gradually transform from conical shapes into rod-like structures. Figure 2d documents the existence of nanoscale grains on the conformally grown AZO facets.

**Figure 3:**
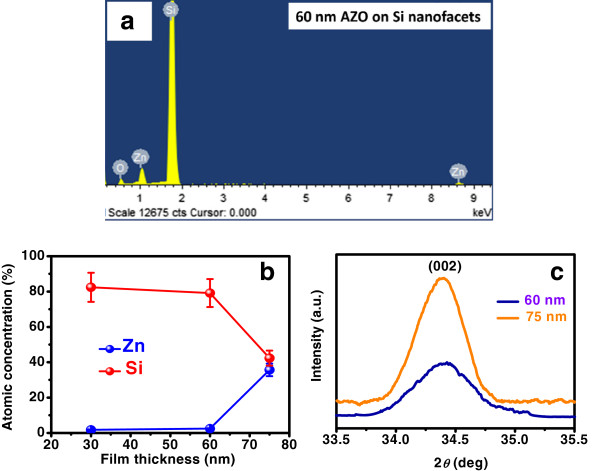
**EDS and XRD study results. (a)** Representative EDS spectrum of 60-nm-thick AZO overlayer grown on Si nanofacets, showing the presence of Si, Zn, and O. **(b)** Plot of atomic concentration versus AZO overlayer thickness obtained from EDS analyses. The solid lines are guide to the eyes. **(c)** X-ray diffractograms of AZO films grown on nanofaceted silicon. The signal corresponding to the 30-nm-thick AZO overlayer is not strong, and therefore, the corresponding diffractogram is not shown here.

The elemental composition of these samples was studied by energy dispersive X-ray spectrometry (EDS) analysis which does not reveal the presence of any metallic impurity in these facets. A representative EDS spectrum corresponding to the 60-nm-thick AZO film on nanofaceted Si is depicted in Figure 3a. Thickness-dependent EDS study demonstrates that concentration of Zn increases with increasing film thickness, while that of silicon decreases rapidly (Figure 3b). Subsequent elemental mapping exhibits Zn-rich apex of the conformally grown AZO faceted structures. Morphological evolution for AZO overlayer of more than 75 nm thick is not presented here since the reflectance minimum goes beyond the spectral range (will be discussed later). Crystalline nature of the AZO overlayers was revealed from XRD studies (Figure 3c), where the appearance of only one peak, in addition to the substrate silicon signal (not shown), can be attributed to the oriented nature of grains. This peak, at all thicknesses, matches well with the (002) reflection of the hexagonal wurzite phase of AZO indicating a preferential growth along the *c*-axis [16]. The average grain size determined from Scherrer's formula is seen to grow bigger with increasing AZO thickness [17]. This corroborates well with the grain size analysis performed on the basis of the SEM studies.

**Figure 4:**
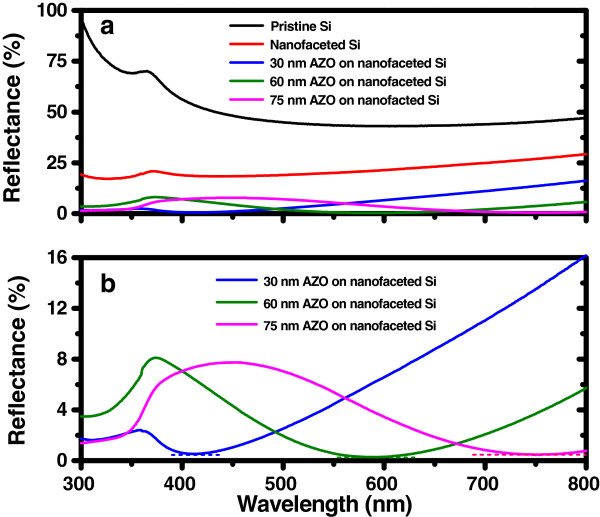
**Surface reflectance spectra. (a)** Reflectance spectra corresponding to pristine Si, nanofaceted Si, and AZO overlayers grown on faceted Si having thicknesses of 30, 60, and 75 nm. **(b)** Reflectance spectra obtained from 30-, 60-, and 75-nm-thick AZO films deposited on faceted Si where the dashed line corresponds to the domain of reflectance minima for different AZO layer thicknesses.

The key result is the change in surface reflectance with increasing AZO thickness on nanofaceted Si templates (Figure 4). In particular, it presents the reflectance data of pristine and faceted silicon along with those obtained from AZO films of varying thicknesses (Figure 3a). Due to the faceted structures, the calculated average residual reflectance [18], over the spectral range of 300 to 800 nm, reduces by 58.5% (compared to that of pristine Si). It is evident from Figure 3a that upon coating the Si template (nanofaceted Si substrate) by a 30-nm-thick AZO film, it exhibits a low average residual reflectance of 6.4%, whereas the conformally grown 60-nm-thick AZO film leads to a further reduction down to 3.1%. However, an increased film thickness of 75 nm causes a nominal increase in the average residual reflectance up to 3.8% which increases further for thicknesses higher than this. A careful observation of the reflectance spectra reveals that the local reflectance minimum of each spectrum (corresponding to different AZO film thicknesses) gets red shifted (Figure 3b). For instance, the 30-nm-thick AZO film shows reflectance below 1% for a spectral range of 385 to 445 nm with a local minimum of approximately 0.5% at 415 nm. Likewise, for the 60-nm-thick overlayer, this range shifts to 530 to 655 nm and the minimum reflectance is found to be approximately 0.3% at 585 nm. Further increase in AZO layer thickness (75 nm) leads to the minimum reflectance of approximately 0.5% at 745 nm. Such shifts in the local minima were previously reported by Boden et al*.*[19] for an antireflective silicon surface. Thus, one can infer that tunable AR property of conformally grown AZO films on nanofaceted Si templates can be achieved by varying the thickness and there exists a critical thickness (60 nm in the present case) which exhibits the best AR performance over the given spectral range (300 to 800 nm).

It may be mentioned that effect of the experimental geometry was tested by subsequent measurement of the surface reflectance after giving a perpendicular rotation to the samples. However, no difference in the reflectance values (within the experimental error) was observed in both cases. To understand this behavior, we calculated the average aspect ratio of the faceted structures (i.e., height/lateral dimension) along *x* and *y* directions which turned out to be 0.25 and 0.24, respectively. It is well known that reflectance depends on the aspect ratio of the surface features [20]. Thus, the observed absence of change in surface reflectance, due to different directions of incident light, can be attributed to the comparable aspect ratio of the faceted structures along *x* and *y* directions.

**Figure 5:**
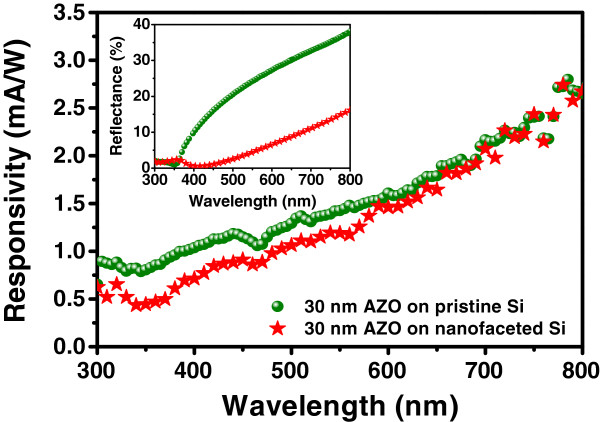
**RT photoresponsivity.** Photoresponsivity spectra of 30-nm-thick AZO overlayer grown on planar and nanofaceted Si in the spectral range of 300 to 800 nm. The inset shows the optical reflectance spectra for these two samples mentioned above.

Figure 5 shows RT photoresponsivity of two sets of samples, *viz.* 30-nm AZO deposited on pristine and faceted silicon. It is observed that the photoresponsivity reduces in the case of the latter one in the projected wavelength range. Different parameters such as short-circuit current densities (*J*_SC_), open-circuit voltages (*V*_OC_), and FF for the above samples are summarized in Table 1 under air mass 0 and 1 sun illumination condition for other AZO thicknesses as well. The FF is defined as FF = (*V*_M_*J*_M_)/ (*V*_OC_*J*_SC_), where *V*_M_*J*_M_ is the maximum power density. From Table 1, one can see that the FF increases by a factor of 2 in the case of AZO overlayer grown on faceted silicon as compared to the one on pristine silicon, whereas *V*_OC_ is found to be half the value obtained from the latter one. In addition, *J*_SC_ becomes 1 order of magnitude higher in the case of AZO-coated faceted silicon, and the same trend is followed for higher AZO thicknesses. From Table 1, it is observed that the FF reaches maximum at 60-nm AZO on faceted silicon (0.361) as compared to others. This improvement in FF can be attributed to the effective light trapping in the visible region in the case of conformally grown AZO films on nanofaceted silicon template [21]. This would ensure the usage of more photogenerated power, leading to an increase in the cell efficiency. Such enhancement in light trapping is found to be directly associated with the enhanced AR property of the same film (inset of Figure 5). However, the reduced *V*_OC_ can be attributed to the existence of defect centers in the native oxide at the AZO/Si interface and ion beam-produced traps on silicon facets. It may be mentioned that AZO/Si heterostructures, in general, yield low FF values and can be improved by using nanofaceted silicon substrates [22]. Thus, our experimental results suggest that besides tunable AR property (Figure 4), FF can also be improved by adjusting the AZO overlayer thickness.

**Table 1:** Different photovoltaic parameters obtained from various AZO overlayer thicknesses grown on silicon substrates

**Sample**	***J***_**SC**_**(mA/cm**^**2**^**)**	***V***_**OC**_**(V)**	***FF***
30-nm AZO on pristine Si^a^	1.24 × 10^-3^	0.133	0.142
30-nm AZO on nanofaceted Si	3.0 × 10^-2^	0.075	0.279
60-nm AZO on nanofaceted Si	5.35 × 10^-2^	0.087	0.361
75-nm AZO on nanofaceted Si	37.57 × 10^-2^	0.055	0.252

^a^Higher AZO thicknesses (beyond 30 nm) deposited on planar silicon substrate did not show any significant photoresponsivity.

Compared to the inverted pyramid approach [23,24], which yields reflectance values between 3% and 5% for an optimized AR coating thickness between 400 and 1,000 nm, our results show a better (by a factor of 10) performance with a smaller (30 to 75 nm) AZO film thickness. Among the available techniques reported in the literature, our novel approach of fabricating faceted nanostructures is simple and can be seamlessly integrated with the modern thin film solar cell technology for better photon harvesting with the help of proper understanding of AR property of AZO films. For a flat surface having an AR overlayer, using Fresnel's reflection formula, we measured the reflectance at different wavelengths. It is observed that with varying film thickness, the position of the reflection minima shifts, while a change in the refractive index modifies the amount of surface reflectance [25]. Although similar trends are quite evident, the experimentally observed average surface reflectance turns out to be much lower over the spectral range under consideration.

In order to explain these results, let us first try to understand the role of the Si template which is practically an ensemble of ion beam-fabricated self-organized conical nanofacets at the top of the Si substrate. It is known that grating on any surface can be used to achieve arbitrary refractive index if the geometry of the grating structures can be tuned. For instance, if we consider a binary grating, its effective refractive index, *n*_eff_, can be expressed as *n*_eff_ = (*n*_1_ - 1)DC + 1, where *n*_1_ is the refractive index of the grating and DC is the duty cycle and is defined as the ratio of the grating line width to the grating period [26]. If the surrounding medium is taken as air and the grating is of the same material as the substrate, the optimized duty cycle (to meet the AR criterion) can be expressed as $\mathrm{DC}=\frac{\sqrt{n_{2}-1}}{n_{2}-1}$ where *n*_2_ is the refractive index of the substrate [26]. Such binary gratings are expected to exhibit the AR property over a very narrow spectral range. This range can be broadened by continuous tuning of the refractive index (*n*_eff_) between the two surrounding media. This would essentially mean a continuous change in DC along the depth (from the apex towards the base of the facets) of the grating lines, which is possible to be achieved by having tapered/conical gratings. When the grating and the substrate materials are the same, the matching of refractive index at the substrate interfaces can exhibit highly improved AR property [27]. This explains the enhanced AR performance observed here for the faceted Si surface formed on the Si substrate. Following the same argument, further improved AR performance is expected due to the conformal growth of an AZO overlayer on nanofaceted Si template. Indeed, the experimental findings confirm the same where increasing AZO thickness leads to a systematic red shift in the reflection minima. However, such small variations in the thickness may not be sufficient to cause any significant difference in depth-dependent change of the effective refractive index for the AZO-coated faceted Si template which corroborates well with the experimentally measured reflectance minima values.

## Conclusions

In conclusion, we show that conformally grown AZO films on ion beam-fabricated self-organized nanofaceted Si templates can work in tandem to yield improved AR performance. It is observed that tunable AR property can be achieved by varying the thickness of AZO overlayer and there exists a critical thickness (60 nm in the present case) which exhibits the best AR performance over the given spectral range (300 to 800 nm). Reduction in surface reflectance for Si templates can be understood in light of gradient refractive index effect arising from a continuous change in the effective refractive index along the depth (from the apex towards the base of the facets) and refractive index matching at the substrate interface because of self-organized nanofaceted Si structures. Following the same argument, further enhancement in the AR performance is observed due to conformal growth of AZO overlayers on Si templates. This is accompanied by a thickness-dependent systematic red shift in the reflection minima. The fabricated AZO/Si heterostructures, both on planar and faceted silicon, show significant photoresponsivity where thickness-dependent fill factor increases by a factor up to 2.5 owing to improved light absorption in the latter case. This study indicates that conformally grown AZO overlayer on nanofaceted silicon may be a promising candidate as AR coatings by optimizing the process parameters.

## Competing interests

The authors declare that they have no competing interests.

## Authors' contributions

TB performed irradiation experiments and data analysis besides writing the manuscript. MK and PKS performed some additional experiments followed by critical data analysis. AK helped in data analysis and contributed in the writing of the manuscript. TS conceived the idea, supervised the research, and incorporated the final corrections into the manuscript. All authors read and approved the final manuscript.
